# Supplementary material for: Genetic background affects the strength of crossover interference in house mice
Source: bioRxiv. 2024 May 30:2024.05.28.596233. Preprint. [Version 1] doi: 10.1101/2024.05.28.596233 (PMC11160618; doi:10.1101/2024.05.28.596233)
Supplement: Supplementary file 1 [file NIHPP2024.05.28.596233v1-supplement-4.pdf]

bioRxiv preprint doi: https://doi.org/10.1101/2024.05.28.596233; this version posted May 31, 2024. The copyright holder for this preprint (which was not certified by peer review) is the author/funder, who has granted bioRxiv a license to display the preprint in perpetuity. It is made available under aCC-BY 4.0 International license.

Supplementary material

Table S1. Sample metadata for $n = 503$ N2 progeny of F1 fathers.

Table S2. All inferred autosomal haplotype segments ($m = 15\,834$) for $n = 503$ mice.

Table S3. Number of autosomal crossovers inferred per individual for $n = 503$ mice.

Raw and processed genotype matrices are available from Figshare.

bioRxiv preprint doi: https://doi.org/10.1101/2024.05.28.596233; this version posted May 31, 2024. The copyright holder for this preprint (which was not certified by peer review) is the author/funder, who has granted bioRxiv a license to display the preprint in perpetuity. It is made available under aCC-BY 4.0 International license.

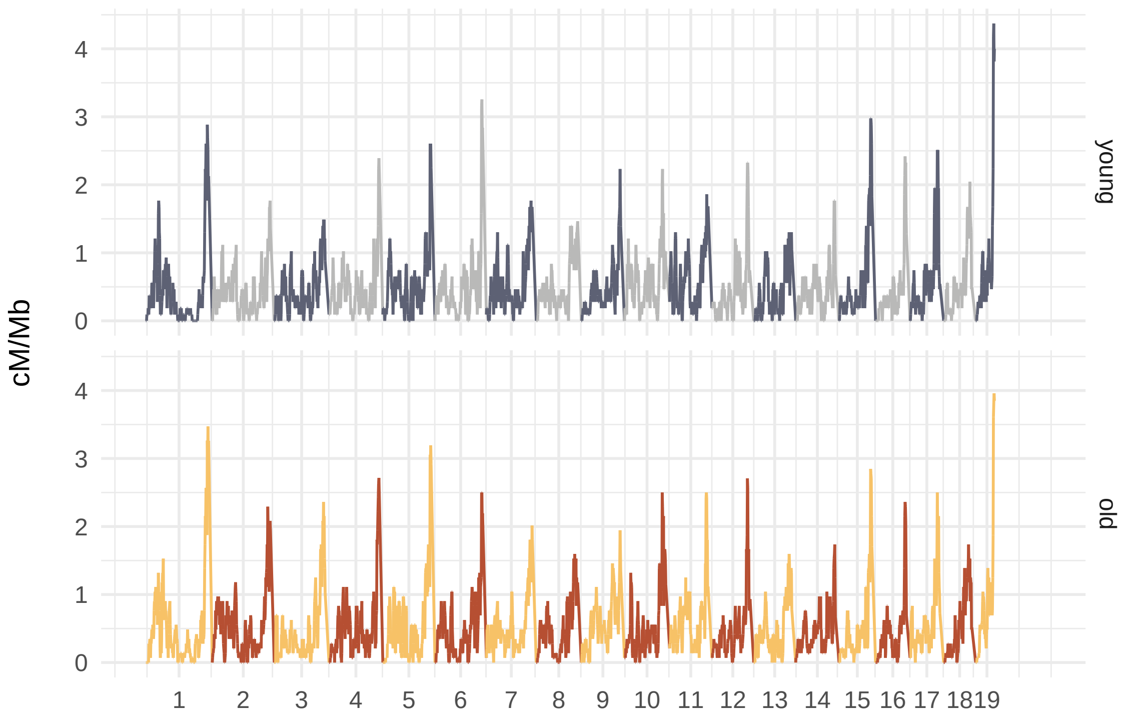

Figure S1: Recombination rate (in cM/Mb) in 5 Mb sliding windows across the autosomes, estimated separately for young and old males.

bioRxiv preprint doi: https://doi.org/10.1101/2024.05.28.596233; this version posted May 31, 2024. The copyright holder for this preprint (which was not certified by peer review) is the author/funder, who has granted bioRxiv a license to display the preprint in perpetuity. It is made available under aCC-BY 4.0 International license.

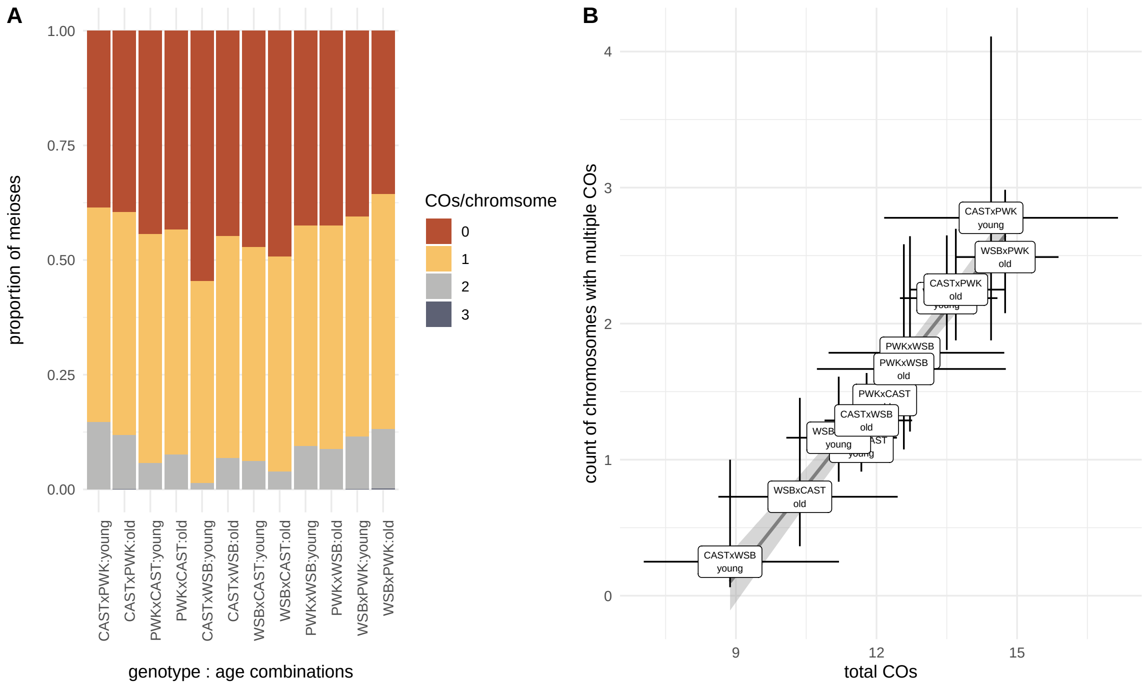

Figure S2: The number of multiply-recombinant chromosomes increases smoothly with recombination rate. (**A**) Distribution of chromosomes by number of crossovers in each genotype-by-age combination. (**B**) Count of chromosomes with multiple crossovers versus total number of crossovers in each genotype-by-age combination. Error bars are 95% confidence intervals from generalized linear model with genotype, age and their interaction as covariates. Solid grey line is a linear fit through the group means.

bioRxiv preprint doi: https://doi.org/10.1101/2024.05.28.596233; this version posted May 31, 2024. The copyright holder for this preprint (which was not certified by peer review) is the author/funder, who has granted bioRxiv a license to display the preprint in perpetuity. It is made available under aCC-BY 4.0 International license.

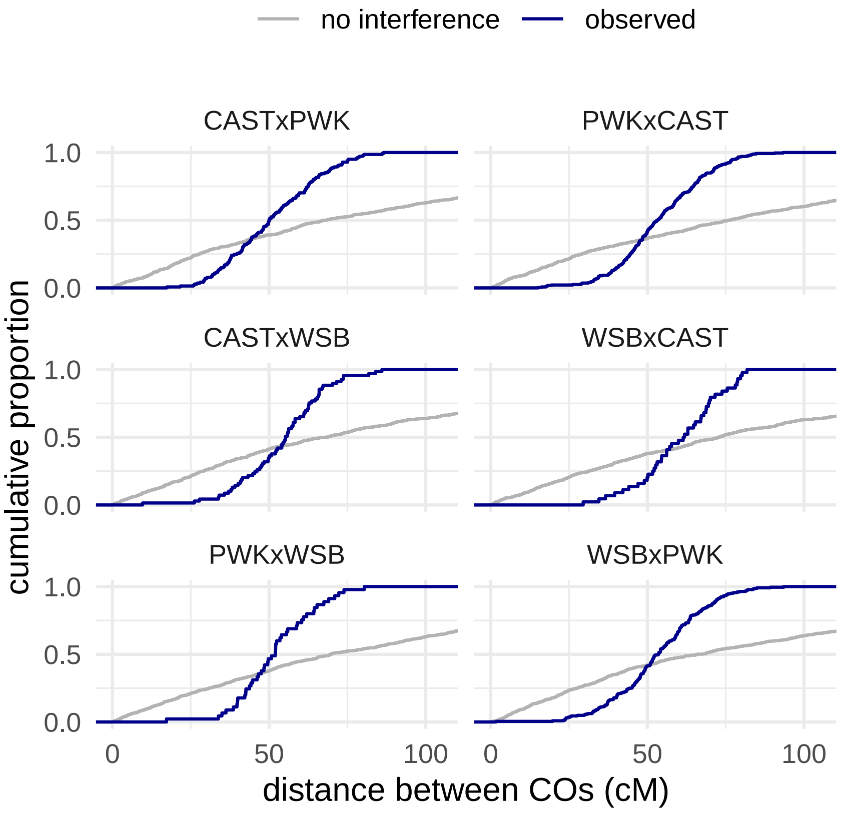

Figure S3: Empirical cumulative distribution of distance between crossovers (COs) on the same chromosome (blue curves), compared to expected distribution without crossover interference.

bioRxiv preprint doi: https://doi.org/10.1101/2024.05.28.596233; this version posted May 31, 2024. The copyright holder for this preprint (which was not certified by peer review) is the author/funder, who has granted bioRxiv a license to display the preprint in perpetuity. It is made available under aCC-BY 4.0 International license.

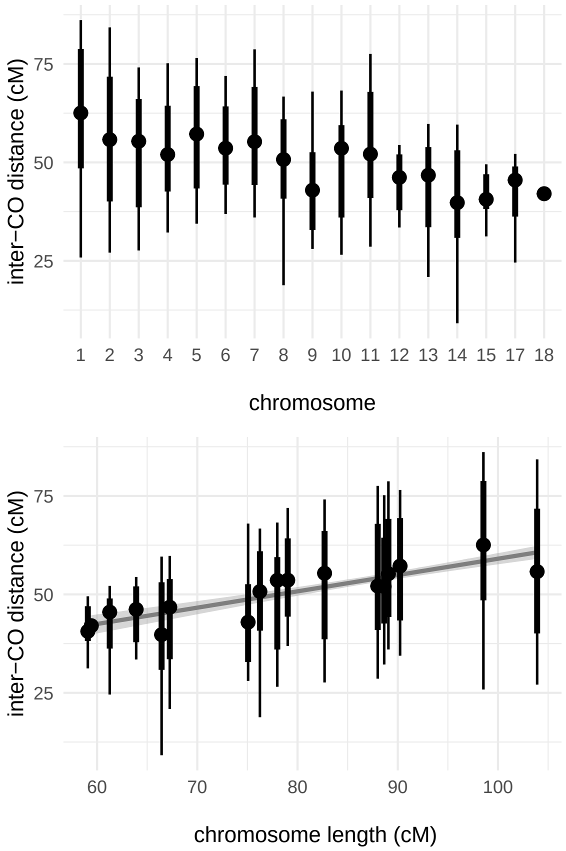

Figure S4: Distribution of inter-crossover distance by chromosome, for chromosomes on which multiply-recombinant progeny were obtained, plotted by chromosome number (**A**) and against total chromosome length (**B**). Solid dots are medians; bars show $2.5\% - 97.5\%$ (thin) and $25\% - 75\%$ (thick) quantile intervals. Solid grey line is a linear fit through the per-chromosome medians.

bioRxiv preprint doi: https://doi.org/10.1101/2024.05.28.596233; this version posted May 31, 2024. The copyright holder for this preprint (which was not certified by peer review) is the author/funder, who has granted bioRxiv a license to display the preprint in perpetuity. It is made available under aCC-BY 4.0 International license.

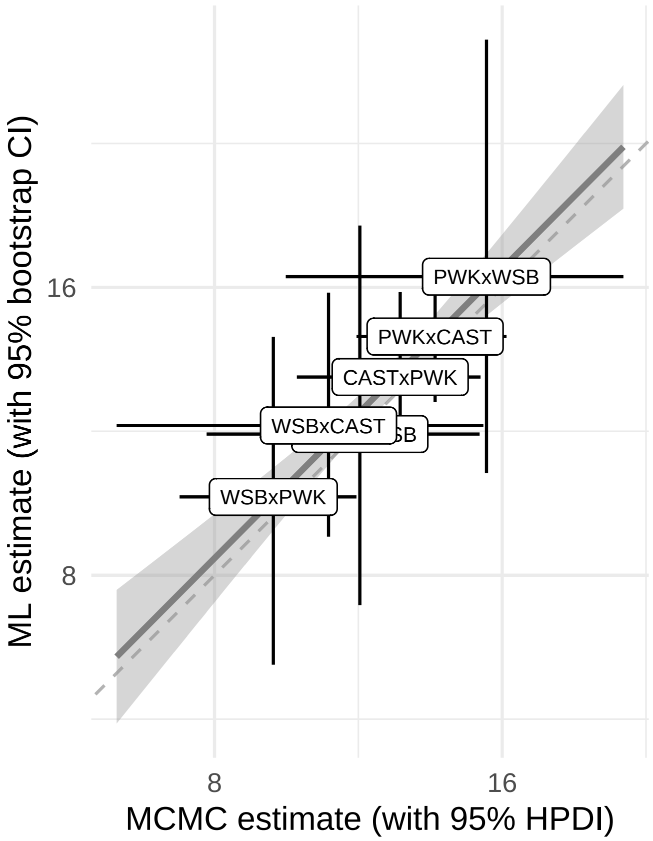

Figure S5: Comparison of group-wise estimates of the interference parameter ν obtained by maximum-likelihood versus joint estimates from Bayesian hierarchical model by MCMC. Solid grey line is a linear fit through the point estimates; dashed grey line has unit slope and passes through the origin.

bioRxiv preprint doi: https://doi.org/10.1101/2024.05.28.596233; this version posted May 31, 2024. The copyright holder for this preprint (which was not certified by peer review) is the author/funder, who has granted bioRxiv a license to display the preprint in perpetuity. It is made available under aCC-BY 4.0 International license.

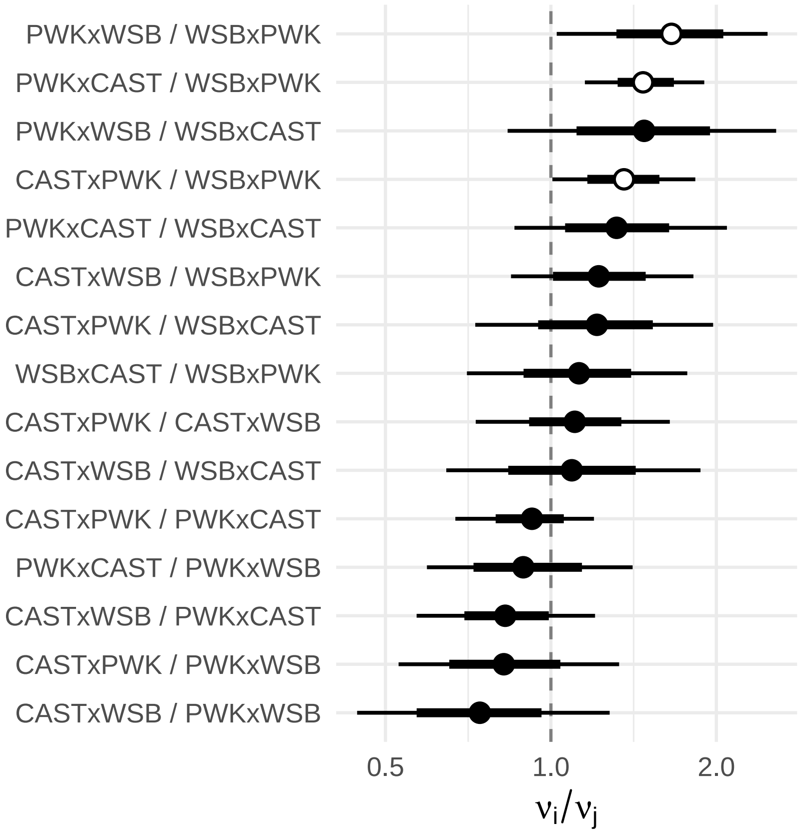

Figure S6: All pairwise contrasts between per-genotype estimates of strength of interference. Dots show posterior means and bars show $2.5\% - 97.5\%$ (thin) and $25\% - 75\%$ (thick) quantile intervals. Open dots indicate comparisons that meet nominal statistical significance; filled dots nominally non-significant. Dashed line gives the null value of 1. Note that the horizontal axis is in log scale.

bioRxiv preprint doi: https://doi.org/10.1101/2024.05.28.596233; this version posted May 31, 2024. The copyright holder for this preprint (which was not certified by peer review) is the author/funder, who has granted bioRxiv a license to display the preprint in perpetuity. It is made available under aCC-BY 4.0 International license.

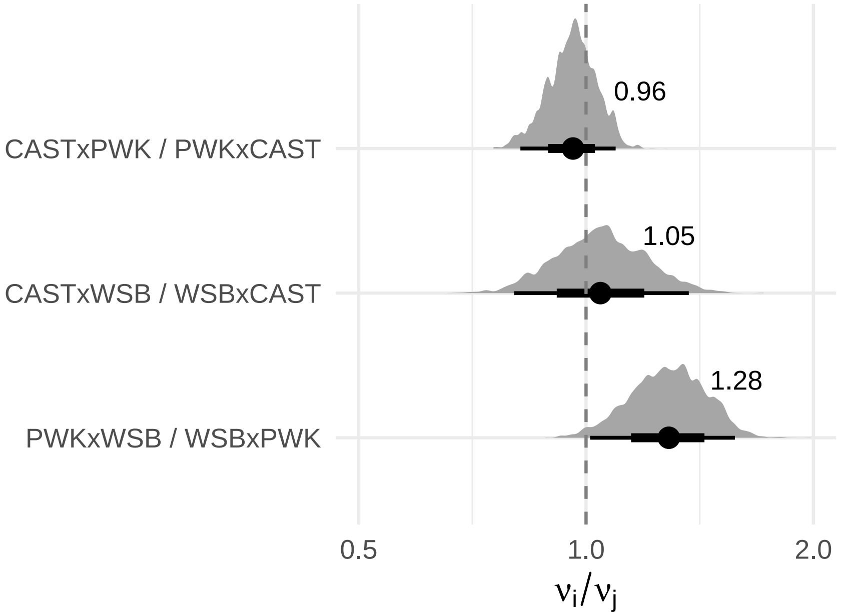

Figure S7: Contrasts between estimates of strength of interference in reciprocal genotypes. Text labels show posterior means. Dashed line gives the null value of 1. Note that the horizontal axis is in log scale.

bioRxiv preprint doi: https://doi.org/10.1101/2024.05.28.596233; this version posted May 31, 2024. The copyright holder for this preprint (which was not certified by peer review) is the author/funder, who has granted bioRxiv a license to display the preprint in perpetuity. It is made available under aCC-BY 4.0 International license.

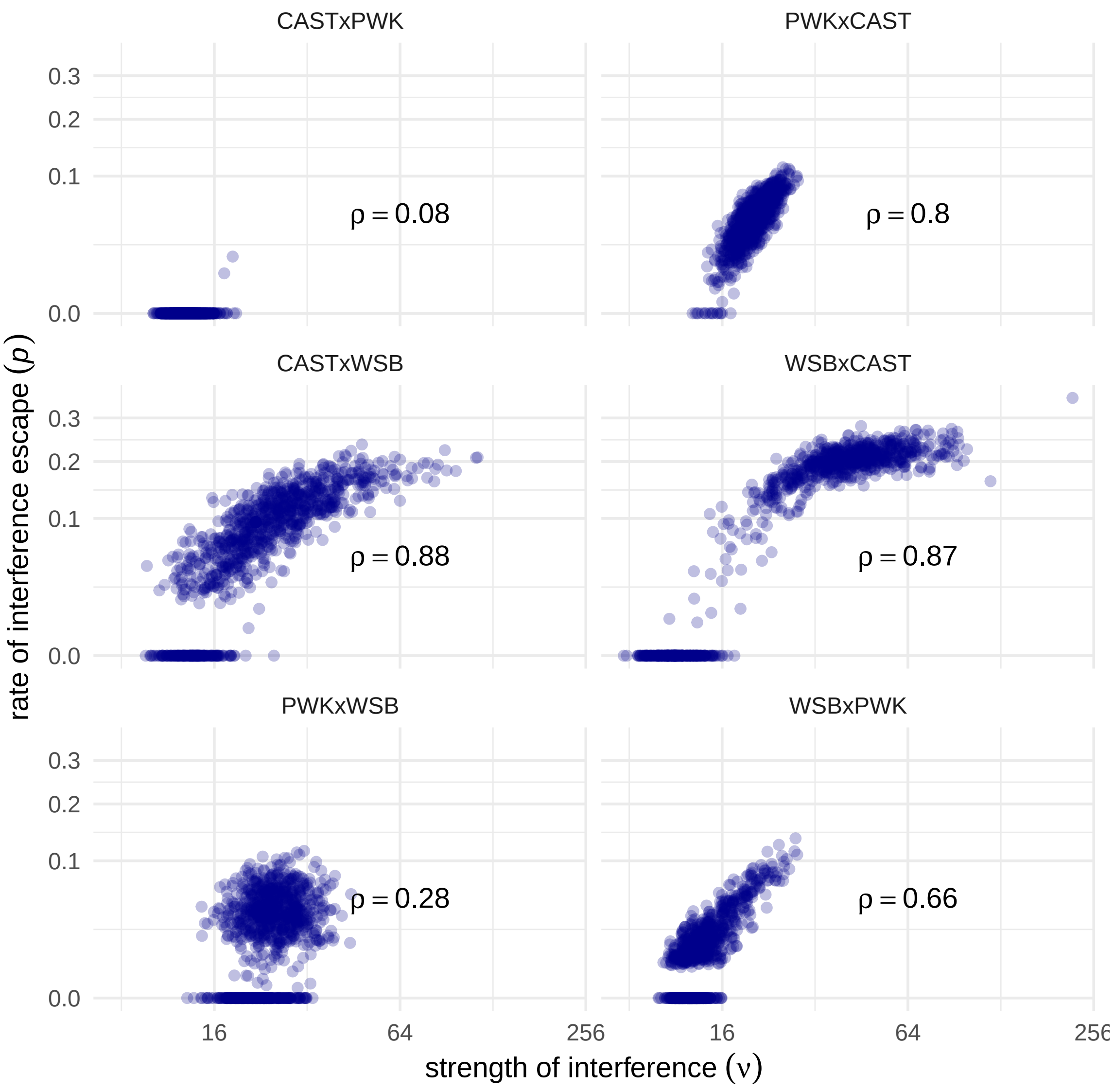

Figure S8: Maximum-likelihood estimates for the strength of interference (ν) and proportion of non-interfering crossovers (p) for 1000 bootstrap replicates of crossover data from six genotypes. Note that horizontal axis is in log scale and vertical axis in square-root scale.

bioRxiv preprint doi: https://doi.org/10.1101/2024.05.28.596233; this version posted May 31, 2024. The copyright holder for this preprint (which was not certified by peer review) is the author/funder, who has granted bioRxiv a license to display the preprint in perpetuity. It is made available under aCC-BY 4.0 International license.

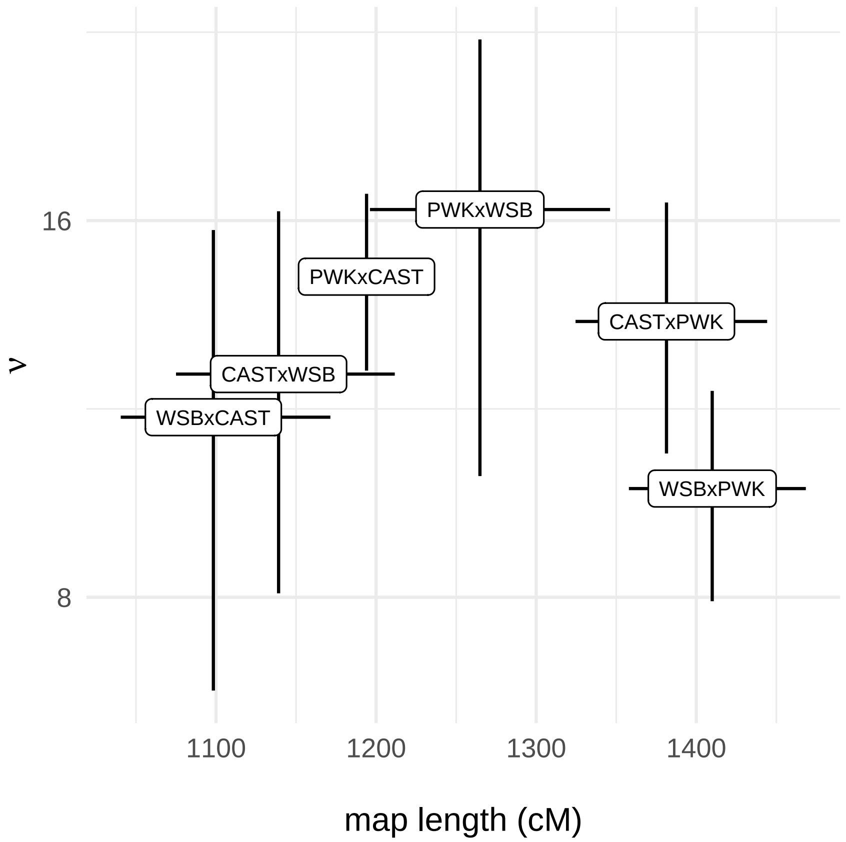

Figure S9: Relationship between length of the recombination map (in cM) and strength of crossover interference (ν). Error bars give 95% confidence bounds, calculated as described for the respective parameters elsewhere.
